# A versatile H5N1-VSV platform for safe influenza virus research applications

**DOI:** 10.1128/jvi.00975-25

**Published:** 2025-08-08

**Authors:** Boopathi Sownthirarajan, Maya Mason, Gayathri Loganathan, Senthamizharasi Manivasagam, Rohit K. Jangra, Gene S. Tan, Daniel R. Perez, Balaji Manicassamy

**Affiliations:** 1 Department of Microbiology and Immunology, University of Iowa 311821 , Iowa City, Iowa, USA; 2 Department of Microbiology and Immunology, Louisiana State University Health Sciences Center-Shreveport 23346, Shreveport, Louisiana, USA; 3 Infectious Diseases, J. Craig Venter Institute 272939 , La Jolla, California, USA; 4 Division of Infectious Diseases and Global Public Health, Department of Medicine, University of California San Diego 196266, La Jolla, California, USA; 5 Wertheim School of Public Health and Human Longevity Science, University of California San Diego 8784, La Jolla, California, USA; 6 Poultry Diagnostic and Research Center, Department of Population Health, College of Veterinary Medicine, University of Georgia 70734, Athens, Georgia, USA; St. Jude Children's Research Hospital, Memphis, Tennessee, USA

**Keywords:** H5N1-VSV, VSV vector, H5N1, influenza virus

## Abstract

The H5N1 strain of influenza A virus (IAV) continues to cause severe infections in a range of avian and mammalian species, including sporadic but concerning cases in humans. There is growing concern that circulating H5N1 strains could lead to widespread human outbreaks. Research with highly pathogenic H5N1 viruses is restricted to Biosafety Level 3 (BSL-3) laboratories. Vesicular stomatitis virus (VSV)-based vaccine vectors expressing heterologous viral proteins from Ebola, SARS-CoV-2, Lassa virus, etc., have previously been shown to be safe and effective in animal models and human clinical trials. Here, we report the development of a recombinant VSV expressing the hemagglutinin (HA) and neuraminidase (NA) genes of H5N1 IAV (H5N1-VSV), which serves as a versatile platform to study various aspects of H5N1 IAV biology. H5N1-VSV replicated robustly to titers comparable to those of the full H5N1 virus in multiple cell lines. In mice, H5N1-VSV vaccination was safe, elicited strong immunity, and conferred protection against a circulating H5N1 strain. Notably, we found that polymorphisms in antigenic site Sa of circulating strains emerged under immune selection pressure in cattle, resembling the evolution of pandemic IAV in humans. These findings suggest that H5N1-VSV can serve as a safe, adaptable platform for influenza research.

## LETTER

**Fig 1:**
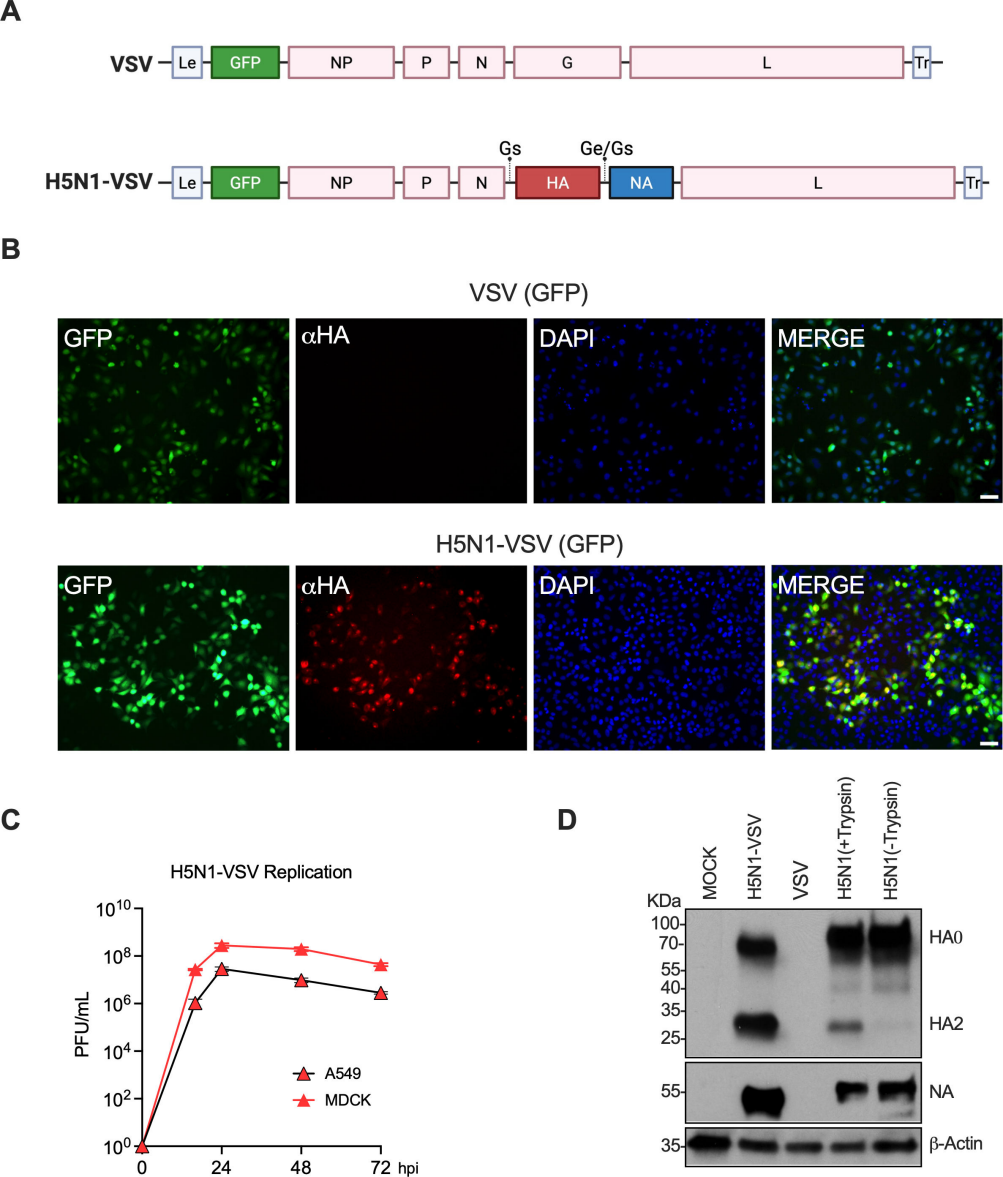
Generation of recombinant VSV expressing HA and NA from the 2024 H5N1. (**A**) Genome organization of VSV-GFP and H5N1-VSV. (**B**) Immunofluorescence of HA and GFP in A549 cells infected with H5N1-VSV. Cells were infected with indicated viruses (MOI = 1), and at 16 hours post infection (hpi), cells were fixed and subjected to immunofluorescence analysis. (**C**) Replication kinetics in A549 and MDCK cells. Cells were infected with H5N1-VSV (MOI = 0.01) and viral loads in the supernatants were measured. Data are shown as mean titer (PFU/mL) of triplicate samples ± SD. (**D**) Western blot for HA and NA in infected MDCK cells. Cells were infected with indicated virus (MOI = 1), and at 16 hpi, cell lysates were collected for Western blot analysis. Low-pathogenic H5N1 is included as control.

To construct a VSV vector expressing H5N1 HA and NA, we replaced the endogenous VSV G ORF with the HA ORF and inserted the NA gene as a separate transcriptional cassette flanked by gene start (GS) and gene end (GE) signals (Fig. 1A, HA/NA based on the A/Michigan/90/2024 H5N1 isolate) (1, 2). To alleviate any potential concerns about recombination or reassortment, we codon-deoptimized the HA and NA sequences and excluded all viral non-coding regions. The VSV backbone also included a GFP reporter as the first gene cassette following the leader RNA, allowing easy visualization of infection by microscopy. Recombinant H5N1-VSV was rescued by co-transfecting 293T cells (pre-infected with vaccinia virus expressing T7 polymerase) with the H5N1-VSV antigenome and helper plasmids encoding the VSV N, P, G, and L proteins (3). Rescue of recombinant virus was confirmed by immunofluorescence for HA and GFP expression (Fig. 1B). H5N1-VSV replicated efficiently in both human lung epithelial cells (A549) and Madin-Darby canine kidney (MDCK) cells, reaching titers of 10⁷–10⁸ PFU/mL (plaque-forming units: PFU; Fig. 1C) (4). Expression of HA and NA in infected MDCK cells was confirmed by Western blot (anti-HA2 antibody, NR-4539, BEI Resources; anti-NA antibody Reference (5)). In addition, HA_0_ was properly cleaved into HA1 and HA2 at the multibasic site (Fig. 1D). As controls, lysates from MDCK cells infected with a low-pathogenic H5N1 strain (A/Texas/37/2024, H5N1/Tx24) were included. These results confirm the successful generation of recombinant H5N1-VSV.

**Fig 2:**
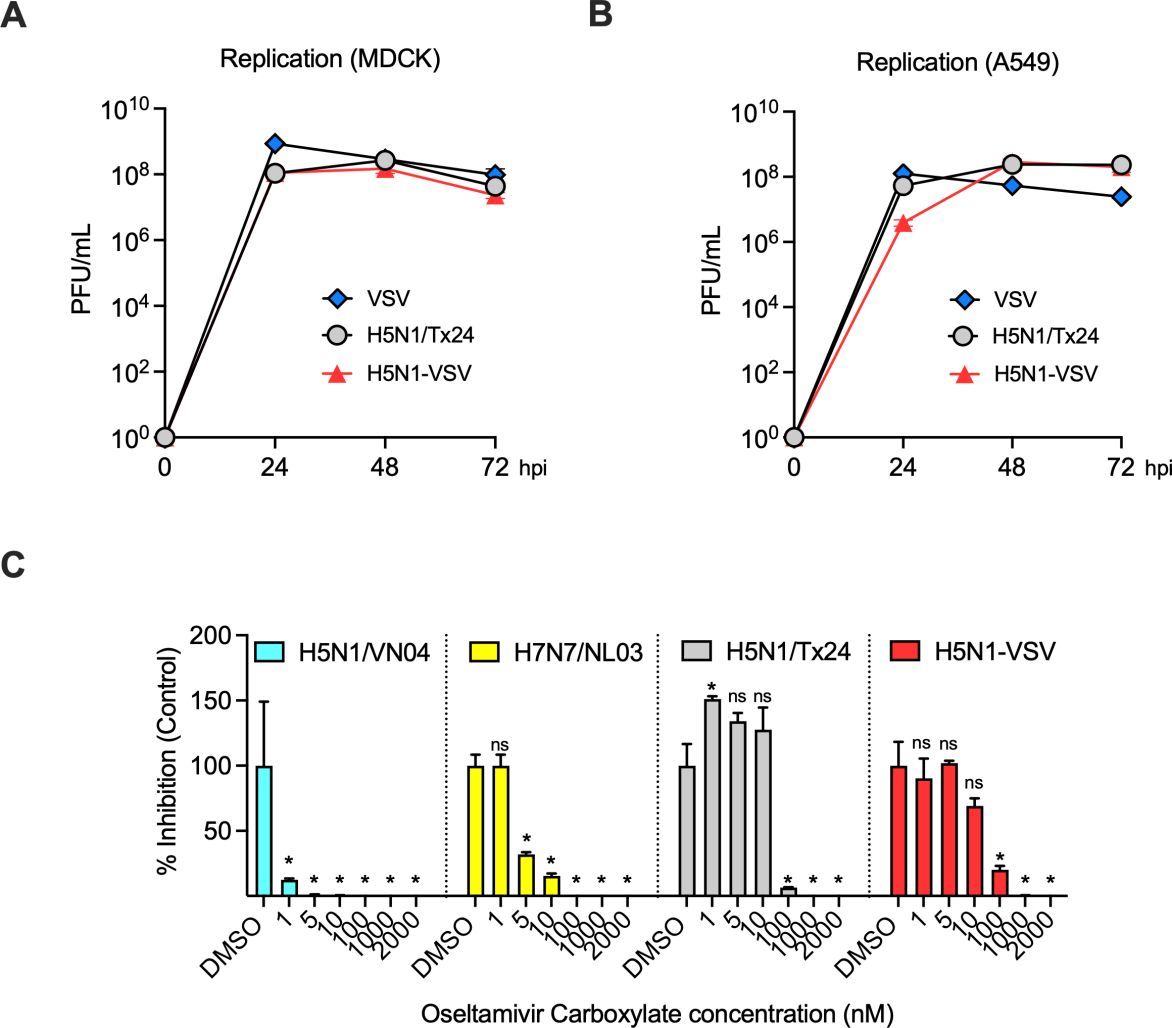
Replication kinetics and oseltamivir sensitivity of H5N1-VSV. (A and B) Replication of H5N1-VSV, H5N1/Tx24, and VSV in A549 and MDCK cells. Cells were infected with indicated viruses (MOI = 0.01), and at various times post-infection, viral loads in the supernatants were measured. Data are shown as mean titer (PFU/mL) of triplicate samples ± SD. (C) Sensitivity of various IAV strains to oseltamivir carboxylate. A549 cells were infected with indicated viruses (MOI = 0.01) and incubated with varying concentrations of oseltamivir carboxylate. At 48 hpi, viral loads in the supernatants were measured. Data are shown as mean percentage inhibition relative to DMSO control of triplicate samples ± SD. Statistical significance was determined by one-way analysis of variance (ANOVA). ∗ denotes *P* < 0.05 or lower, and ns, non-significant.

Next, we compared the replication kinetics of H5N1-VSV with the low-pathogenic H5N1/Tx24 strain in A549 and MDCK cells. H5N1-VSV replicated to similar levels as H5N1/Tx24 and VSV-GFP with viral titers exceeding 10⁸ PFU/mL (Fig. 2A and B). As a recent report indicated that a human isolate of bovine H5N1 (2024) strain shows reduced sensitivity to the neuraminidase inhibitor oseltamivir (Tamiflu), we assessed replication of different IAV strains in A549 cells in the presence of oseltamivir carboxylate (6). Older H5N1 (A/Vietnam/1203/2004; H5N1/VN04) and H7N7 (A/Netherlands/219/2003; H7N7/NL03) strains showed marked sensitivity at lower drug concentrations (Fig. 2C). In contrast, both H5N1-VSV and H5N1/Tx24 showed reduced sensitivity, with inhibition occurring only at higher concentrations. Together, these results support the use of H5N1-VSV as a surrogate model to study circulating H5N1 strains.

**Fig 3:**
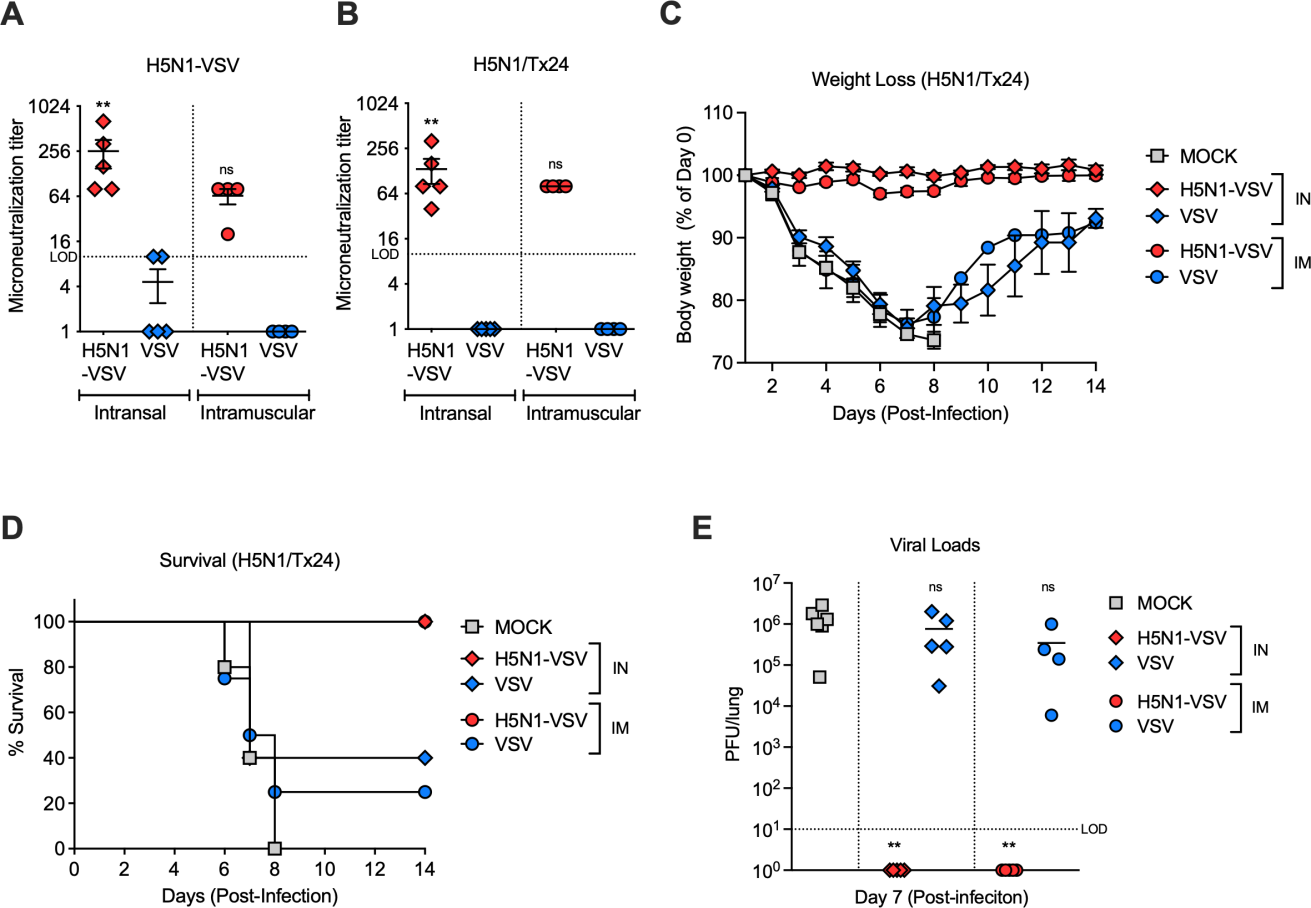
Protective efficacy of H5N1-VSV vaccination in mice. (A and B) Neutralization titers in the sera post-vaccination. Sera from vaccinated mice were treated with RDE-II prior to use in neutralization assays. (C through E) Protection in vaccinated mice following lethal H5N1 challenge: (C) weight loss (*n* = 4–5), (D) survival (*n* = 4–5), and (E) lung titers (*n* = 4–6). Statistical significance was determined by ANOVA. ∗ denotes *P* < 0.05, **P < 0.01, and ns, non-significant.

As VSV-based Ebola vaccines have proven effective in humans, we next evaluated whether H5N1-VSV could protect mice from a low pathogenic H5N1/Tx24 challenge (7, 8). C57BL/6J mice were vaccinated intranasally or intramuscularly with H5N1-VSV and boosted 2 weeks later (doses of 1.25 × 10^7^ PFU). Mice vaccinated with VSV-GFP served as the control group. Four weeks after the initial vaccination, serum was collected for neutralization assays against H5N1-VSV and H5N1/Tx24. Mice vaccinated with H5N1-VSV via either route showed high-titer neutralizing antibodies in the serum as compared to VSV-GFP vaccinated mice (Fig. 3A and B). Upon lethal challenge with 10 LD₅₀ of H5N1/Tx24 (Dose = 2370 PFU), all of the H5N1-VSV vaccinated mice survived without displaying any weight loss or clinical signs of infection (Fig. 3C and D). This protection correlated with an undetectable virus in lung tissues at day 7 post-challenge (Fig. 3E). In contrast, the majority of VSV-GFP vaccinated mice and all of the mock-vaccinated mice succumbed to H5N1/Tx24 challenge and showed high lung viral loads. These results demonstrate that the H5N1-VSV vaccine is safe and elicits strong protective immunity against a circulating H5N1 strain.

**Fig 4:**
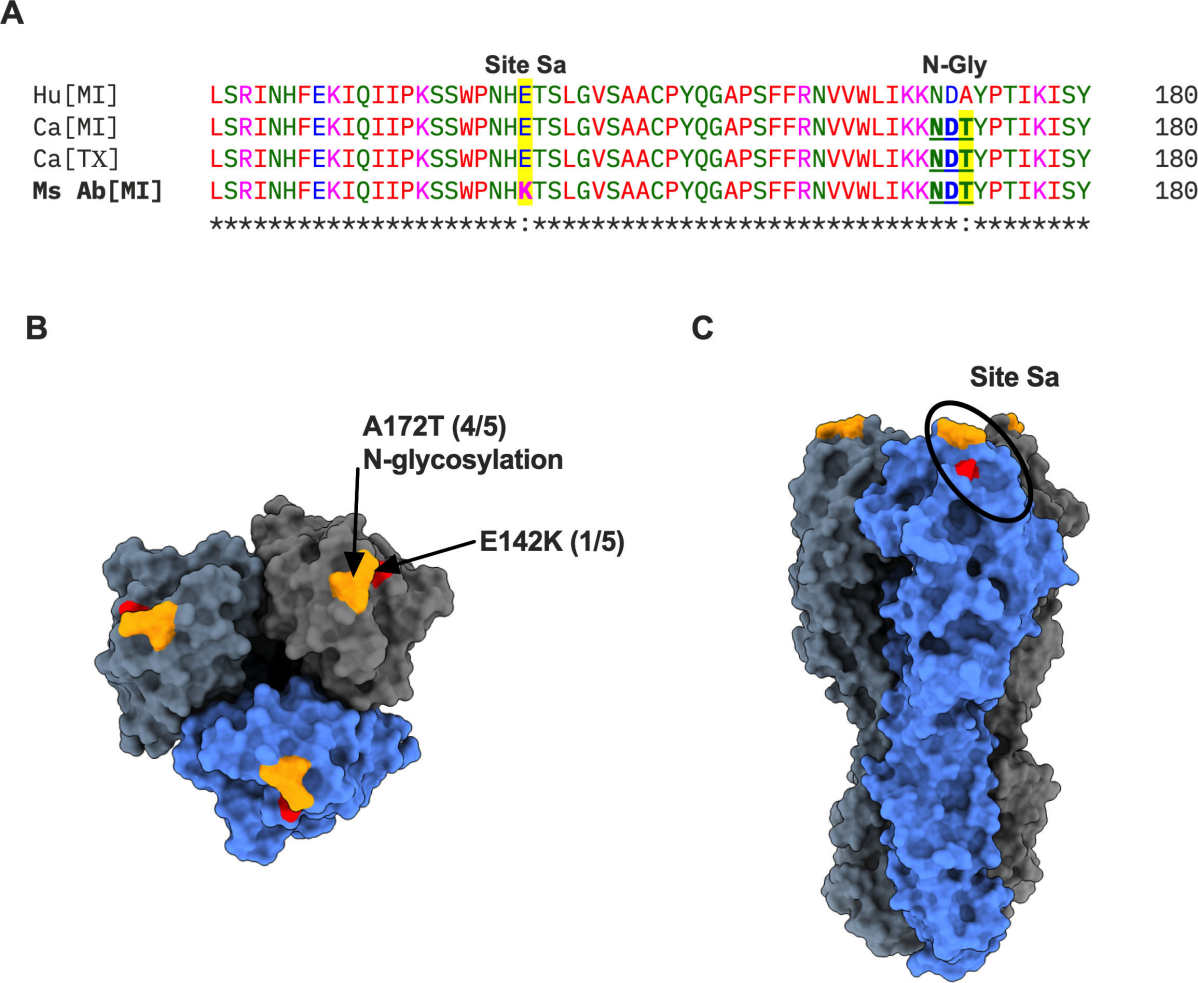
Immune selection at antigenic site Sa in cattle H5N1 isolates. (A) HA sequence comparison of currently circulating H5 isolates and neutralization escape mutants. Hu—human, Ca—cattle, and Ms Ab—mouse polyclonal sera selected variant. GeneBank sequences of indicated strains are Hu[MI]: XBE32674.1, Ca[MI]: XFE99892.1, and Ca[TX]: XAJ06472.1. (B and C) Structural modeling of site Sa mutations on HA trimer. (B) Top view. At passage 5, A172T mutation was present in four out of five independent replicates, and E142K mutation was present in one replicate. (C) Side view.

IAV evolution in humans is often associated with the accumulation of immune-driven mutations in major HA antigenic sites, including the acquisition of N-linked glycosylation sequon (N-X-S/T) (9, 10). We had previously shown that descendants of the 1918 H1N1 virus acquired N-glycosylation at antigenic site Sa upon circulation in humans (11). Interestingly, sequence analysis of circulating H5N1 strains revealed an A172T mutation in cattle isolates, which introduces a potential N-glycosylation site (Fig. 4A). To determine whether this A172T change is due to immune pressure, we passaged H5N1-VSV in MDCK cells in the presence of immune serum (1:100 dilution) from vaccinated mice (*n* = 5 independent replicates). At passage 5, we observed robust H5N1-VSV replication in all five replicates, suggesting the potential emergence of escape variants. Bulk sequencing of viral RNA from passage 5 supernatants revealed that four of five independent replicates acquired the A172T mutation (NDA→NDT), while one had an E142K mutation, both occurring within antigenic site Sa. This remarkable finding further validates the H5N1-VSV vector as a safe and effective tool for investigating antigenic drift in influenza viruses. Critically, it highlights that continued H5N1 circulation in cattle promotes this drift, increasing the risk of new strains emerging with an altered host range—a scenario with significant public health consequences. It should be noted that during passaging of H5N1-VSV, we observed the emergence of mutants with a truncated HA tail (~13 amino acid deletion), as recently reported (12). These findings strongly suggest that immune selection is a key driver of H5N1 IAV evolution within cattle. As human IAV strains initially escape preexisting immunity through antigenic drift at the Sa site, it is likely that H5N1 strains carrying the A172T mutation will evade immunity induced by vaccines based on ancestral H5 strains, underscoring the importance of considering variability at the Sa antigenic site when selecting future vaccine strains.

In summary, our study demonstrates that the H5N1-VSV construct is a powerful and safe tool for studying contemporary H5N1 viruses. This platform allows for research outside of BSL-3 conditions and can support vaccine development, viral evolution studies, and antiviral screening.

## References

[1] Liu G, Cao W, Salawudeen A, Zhu W, Emeterio K, Safronetz D, Banadyga L. 2021. Vesicular stomatitis virus: from agricultural pathogen to vaccine vector. Pathogens 10:1092. doi:10.3390/pathogens10091092 34578125 PMC8470541

[2] Roberts A, Buonocore L, Price R, Forman J, Rose JK. 1999. Attenuated vesicular stomatitis viruses as vaccine vectors. J Virol 73:3723–3732. doi:10.1128/JVI.73.5.3723-3732.1999 10196265 PMC104148

[3] Whitt MA. 2010. Generation of VSV pseudotypes using recombinant ΔG-VSV for studies on virus entry, identification of entry inhibitors, and immune responses to vaccines. J Virol Methods 169:365–374. doi:10.1016/j.jviromet.2010.08.006 20709108 PMC2956192

[4] Heaton NS, Leyva-Grado VH, Tan GS, Eggink D, Hai R, Palese P. 2013. In vivo bioluminescent imaging of influenza a virus infection and characterization of novel cross-protective monoclonal antibodies. J Virol 87:8272–8281. doi:10.1128/JVI.00969-13 23698304 PMC3719835

[5] Wohlbold TJ, Nachbagauer R, Xu H, Tan GS, Hirsh A, Brokstad KA, Cox RJ, Palese P, Krammer F. 2015. Vaccination with adjuvanted recombinant neuraminidase induces broad heterologous, but not heterosubtypic, cross-protection against influenza virus infection in mice. mBio 6:e02556. doi:10.1128/mBio.02556-14 25759506 PMC4453582

[6] Gu C, Maemura T, Guan L, Eisfeld AJ, Biswas A, Kiso M, Uraki R, Ito M, Trifkovic S, Wang T, Babujee L, Presler R, Dahn R, Suzuki Y, Halfmann PJ, Yamayoshi S, Neumann G, Kawaoka Y. 2024. A human isolate of bovine H5N1 is transmissible and lethal in animal models. Nature 636:711–718. doi:10.1038/s41586-024-08254-7 39467571 PMC12629513

[7] Henao-Restrepo AM, Camacho A, Longini IM, Watson CH, Edmunds WJ, Egger M, Carroll MW, Dean NE, Diatta I, Doumbia M, et al.. 2017. Efficacy and effectiveness of an rVSV-vectored vaccine in preventing Ebola virus disease: final results from the Guinea ring vaccination, open-label, cluster-randomised trial (Ebola Ca Suffit!). Lancet 389:505–518. doi:10.1016/S0140-6736(16)32621-6 28017403 PMC5364328

[8] Meakin S, Nsio J, Camacho A, Kitenge R, Coulborn RM, Gignoux E, Johnson J, Sterk E, Musenga EM, Mustafa SHB, Epicentre M, Finger F, Ahuka-Mundeke S. 2024. Effectiveness of rVSV-ZEBOV vaccination during the 2018-20 Ebola virus disease epidemic in the Democratic Republic of the Congo: a retrospective test-negative study. Lancet Infect Dis 24:1357–1365. doi:10.1016/S1473-3099(24)00419-5 39178866

[9] Altman MO, Angeletti D, Yewdell JW. 2018. Antibody immunodominance: the key to understanding influenza virus antigenic drift. Viral Immunol 31:142–149. doi:10.1089/vim.2017.0129 29356618 PMC5863095

[10] York IA, Stevens J, Alymova IV. 2019. Influenza virus N-linked glycosylation and innate immunity. Biosci Rep 39:BSR20171505. doi:10.1042/BSR20171505 30552137 PMC6328934

[11] Medina RA, Stertz S, Manicassamy B, Zimmermann P, Sun X, Albrecht RA, Uusi-Kerttula H, Zagordi O, Belshe RB, Frey SE, Tumpey TM, García-Sastre A. 2013. Glycosylations in the globular head of the hemagglutinin protein modulate the virulence and antigenic properties of the H1N1 influenza viruses. Sci Transl Med 5:187ra70. doi:10.1126/scitranslmed.3005996 PMC3940933 23720581

[12] Robinson-McCarthy LR, Zirckel KE, Simmons HC, Le Sage V, McCarthy KR. 2025. A replicating recombinant vesicular stomatitis virus model for dairy cattle H5N1 influenza virus glycoprotein evolution. J Virol 99:e0038925. doi:10.1128/jvi.00389-25 40464562 PMC12282150

